# Case Report: Severe Rashes Associated With Fruquintinib in a Patient With Metastatic Colorectal Cancer

**DOI:** 10.3389/fonc.2021.688231

**Published:** 2021-07-05

**Authors:** Yefei Shu, Song Zheng

**Affiliations:** ^1^ Department of Medical Oncology, Affiliated Hangzhou Cancer Hospital, Zhejiang University School of Medicine, Hangzhou, China; ^2^ Department of Medical Oncology, Affiliated Hangzhou First People’s Hospital, Zhejiang University School of Medicine, Hangzhou, China

**Keywords:** colorectal cancer, fruquintinib, rash, vascular endothelial growth factor receptor, case report

## Abstract

Metastatic colorectal cancer (mCRC) is a common and high-risk malignant tumor. Fruquintinib is a novel small-molecule compound with high selective inhibition of vascular endothelial growth factor (VEGF) receptor (VEGFR) for mCRC for which second-line or higher standard chemotherapy has been ineffective. A female patient with mCRC developed severe rashes after 2 weeks of taking fruquintinib. Considering the relationship between fruquintinib and the rashes, she discontinued taking the drug, and her condition improved. Although fruquintinib has shown good safety and manageable toxicity in previous trials, the patient in the present case developed severe rashes after 2 weeks of taking fruquintinib. The common skin reactions of hand and foot are erythema and paresthesia of hand and foot. Because few people have reported a severe rash caused by fruquintinib, which is different from the common hand foot skin reaction. We hope the case attracts the attention of oncologists.

## Introduction

Colorectal cancer ranks third and second in the incidence rate of male and female malignancies respectively all over the world (1). There are approximately 1.36 million new patients with colorectal cancer and nearly 700,000 related deaths every year, which is a huge global challenge. In China, the number of new cases is 376,000 every year, and this number continues to grow. In approximately 50% of all cases, colorectal cancer may eventually develop into mCRC or advanced colorectal cancer. After failure of second-line standard treatment, effective treatments of mCRC are limited, and some patients have good resilience and a strong desire for survival.

Fruquintinib is a novel small-molecule compound with high selective inhibition of vascular endothelial growth factor (VEGF) receptor (VEGFR) for mCRC for which second-line or higher standard chemotherapy has been ineffective (2). Although fruquintinib has shown the advantages of a strong effect, low toxicity, and good tolerance for colorectal cancer, it is associated with some unrecognized adverse reactions.

## Case Report

**Figure 1:**
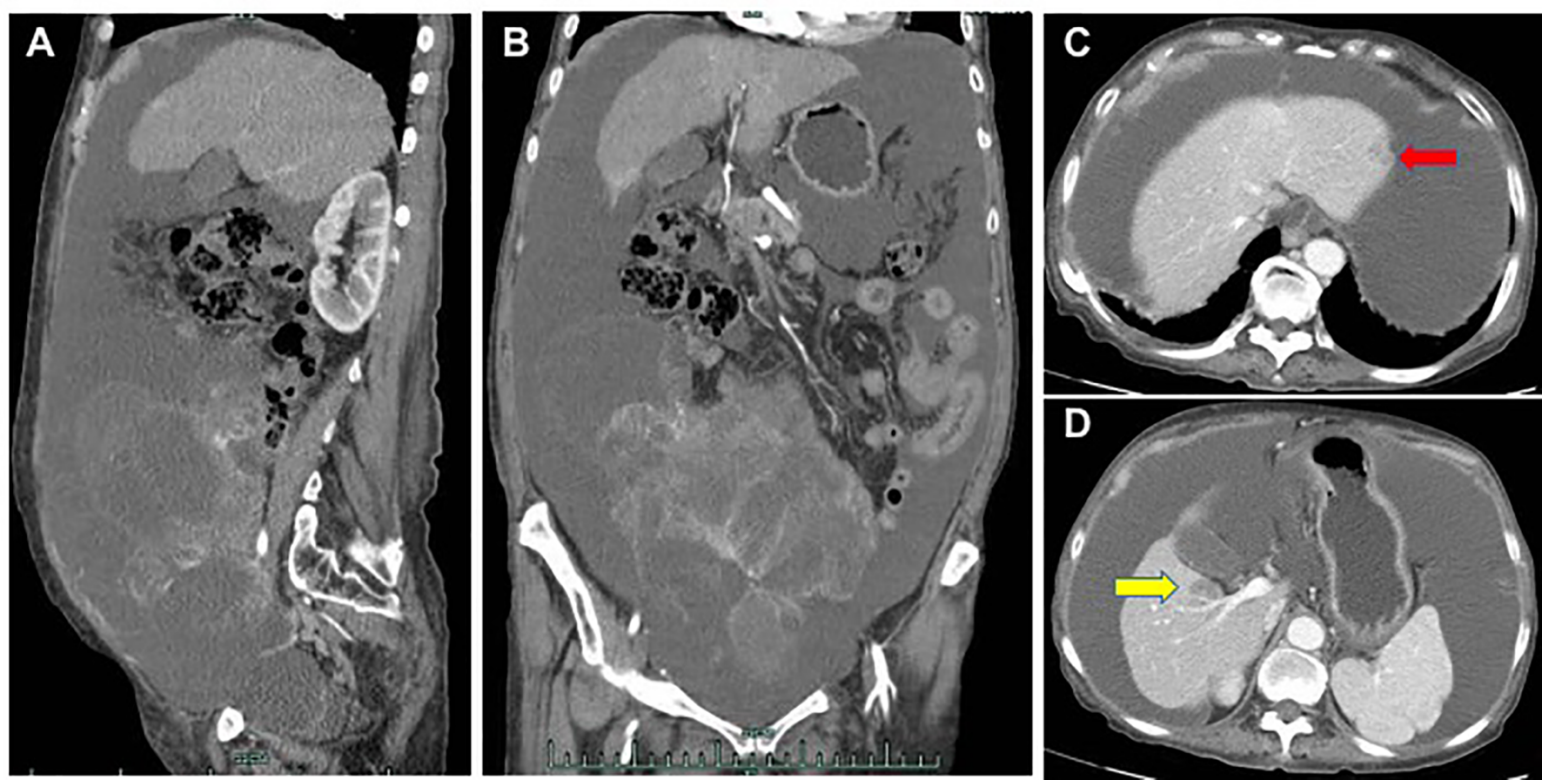
On December 17, 2020, the whole abdominal enhanced CT in our hospital showed a large number of peritoneal effusion, multiple metastases of omentum and mesentery **(A, B)**, liver II metastases (**C**, red arrow) and V metastases (**D**, yellow arrow).

**Figure 2:**
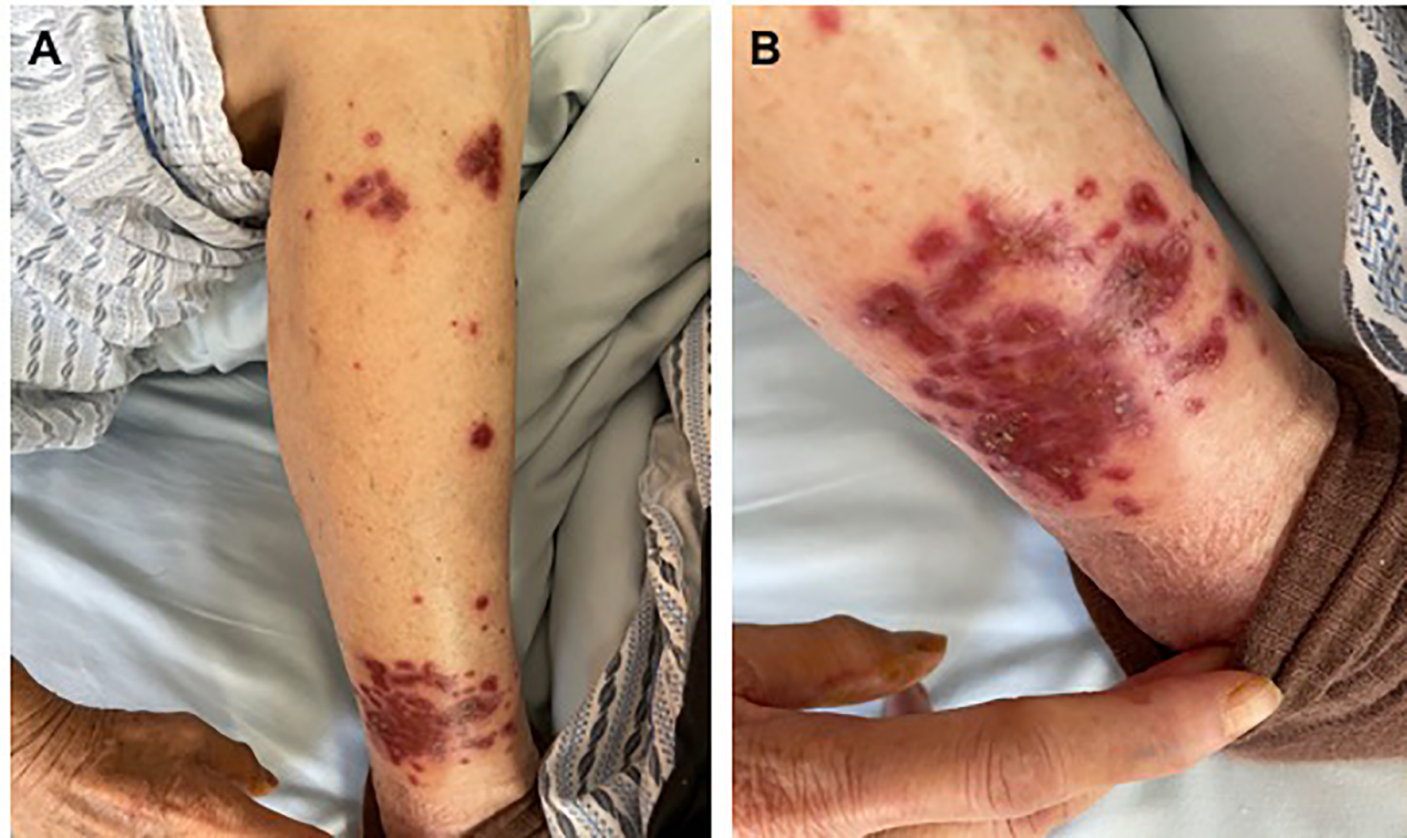
**(A, B)** The severe rashes after receiving fruquintinib for approximately 2 weeks.

**Figure 3:**
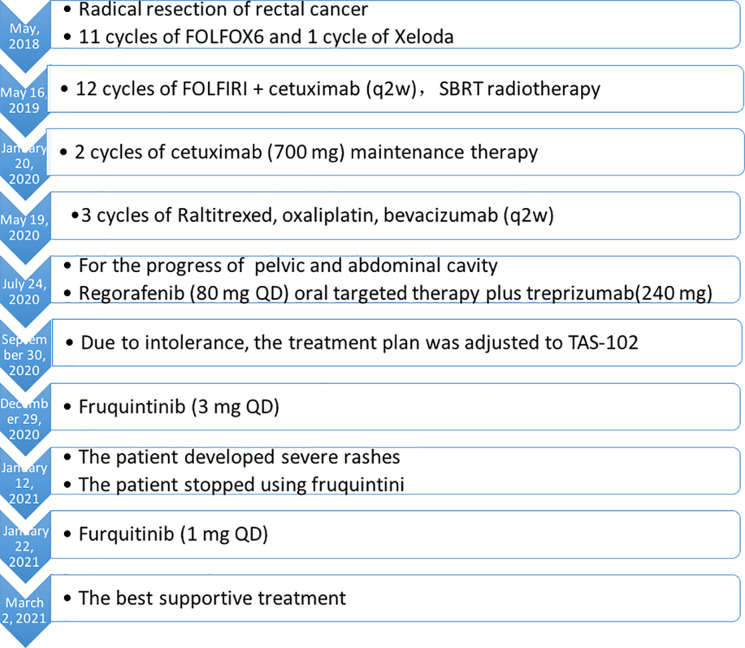
The case timeline.

A 71-year-old woman was admitted to hospital due to increased stool frequency and abdominal discomfort. She had no family history of cancer or drug allergies. She underwent radical resection of rectal cancer in May 2018. Postoperative pathology showed that rectal moderately differentiated adenocarcinoma was 3.5 × 2.5 cm with negative margin. The depth of ulcer was full-thickness infiltration, involving serosa. One of nine lymph nodes had cancer metastasis. Special examination showed MSH2 (+), MSH6 (+), MLH1 (+), PMS2 (+), CDX2 (+). According to the Union for International Cancer Control/American Joint Committee on Cancer tumor–node–metastasis staging system (8th Edition, 2017), the postoperative pathological stage was stage IIIB (T3N1M0). The patient did not receive preoperative and postoperative radiotherapy. Eleven cycles of the FOLFOX6 regimen and one cycle of Xeloda were administered after the surgery, and the patient was then followed up regularly. In May 2019, intrahepatic nodules and peri-intestinal metastasis were considered. Positron emission tomography–computed tomography (CT) showed ascending-colon mesangial and omental nodules, liver S4 and S2 nodules, with increased fluorodeoxyglucose metabolism. Gene detection indicated she harbored wild-type *KRAS*/*NRAS*/*BRAF*, and microsatellite instability detection indicated microsatellite stability (MSS). Therefore, from May 16, 2019, FOLFIRI combined with cetuximab was administered for 12 courses, and from January 20, 2020, cetuximab (700 mg) was administered for two courses as maintenance treatment. When liver metastasis occurred, stereotactic body radiation therapy was performed in other hospital. The plan of radiotherapy is unknown. From May 19, 2020, three courses of raltitrexed, oxaliplatin, bevacizumab (q2w) were administered. On July 21, 2020, pelvic and abdominal cavity CT was performed to evaluate disease progression. Although the patient was a MSS colorectal patient, based on the regonivo study (3) with ORR 36% and PFS 7.9 months and her strong desire for survival, she tried small molecule targeted drugs combined with immunotherapy. From July 24, 2020, regorafenib (80 mg QD) oral targeted therapy plus treprizumab (240 mg) immunotherapy were administered. On September 30, 2020, because of fatigue, hand and foot skin reaction, and loss of appetite, the treatment plan was adjusted to TAS-102. Then, physical examination revealed a large amount of ascites. On December 17, 2020, the whole abdominal enhanced CT in our hospital showed liver II and V metastases considered, a large number of peritoneal effusion, and multiple metastases of omentum and mesentery (**Figure 1**). The disease progressed; therefore, the treatment was changed to fruquintinib. On January 12, 2021, the patient developed severe rashes after receiving fruquintinib (3 mg qd) for approximately 2 weeks (**Figures 2A, B**). The patient developed severe rashes (Common Terminology Criteria for Adverse Events grade 3) in both lower limbs, which affected the patient’s quality of life. Blood routine and coagulation function were basically normal. A dermatology consultation revealed dark purple infiltrative rashes scattered on both lower limbs and pustules observed locally. Betamethasone (1 ml, intramuscular injection), chlorphenamine maleate (10 mg, intramuscular injection), mupirocin ointment (0.2 g, bid for external use), and mometasone furoate cream (5 mg, QN for external use) were recommended. The dermatologist diagnosed these rashes as drug-induced rashes. Then the patient discontinued taking fruquintinib due to severe rashes. The severe rashes disappeared gradually after discontinuation of fruquintinib for 1 week. On January 22, 2021, the patient began to reduce the use of fruquintinib (1 mg QD), and there was no recurrence of rashes. On March 2, 2021, the intraperitoneal tumor was found to have progressed; therefore, fruquintinib was discontinued. In the follow-up, the patient is receiving the best supportive treatment. The case timeline is presented in **Figure 3**.

Informed written consent was obtained from the patient prior to publication of this report and ethical approval was given by Hangzhou Cancer Hospital.

## Discussion

Fruquintinib is a novel small molecule compound with high selective inhibition of VEGFR1, -2, and, -3 (4). It inhibits VEGF-induced VEGFR2 phosphorylation, endothelial cell proliferation, and tubule formation (5). The approval of fruquintinib is based on a multicenter, randomized, double-blind, placebo-controlled phase III clinical study, Fresco. The results of the Fresco study showed that the median overall survival of patients with mCRC treated with fruquintinib was 9.3 months, which was 2.7 months longer than that in the placebo group; in addition, the median progression free survival of patients treated with fruquintinib was 3.7 months, which was significantly longer than 1.8 months in the placebo group, with a significant survival benefit (6).

However, in the Fresco study, grades 3 and 4 treatment-emergent adverse events occurred in 61.2% of patients who received fruquintinib. The most common adverse reactions affecting patients in the fruquintinib group were hypertension, proteinuria, hand foot skin reactions, etc., which were related to the target VEGFR, and the prevalence was relatively low, which was also controllable clinically, and most reactions were tolerable (7). Hand foot syndrome (palmar or plantar swelling, or pain or fingertip erythema) is the most common skin adverse reaction to fruquintinib, which is usually mild to moderate (grade 1–2). Fruquintinib also exhibits cross-toxicity with other anti-vascular drugs (such as bevacizumab), including hypertension, proteinuria, bleeding. A meta-analysis has shown that fruquintinib exhibits less toxicity among all-grade toxicities when compared with that of regorafenib (8).

The common skin reactions of hand and foot are erythema and paresthesia of hand and foot. Because few people have reported a severe rash caused by fruquintinib, which is different from the common hand foot skin reaction. The mechanism of fruquintinib induced rash is not very clear. We think it may be related to the inhibition of VEGFR2/3 phosphorylation by fruquintinib, thus inhibiting the proliferation and lumen formation of endothelial cells. The specific mechanism of severe skin rash caused by fruquintinib deserves further exploration.

## Conclusion

Cancer therapy faces the challenge of handling a double-edged sword. Fruquintinib brings not only clinical benefits, but also some adverse reactions. How to manage fruquintinib to maximize the therapeutic effect and avoid adverse reactions as much as possible is a problem we need to explore.

## Data Availability Statement

The raw data supporting the conclusions of this article will be made available by the authors, without undue reservation.

## Ethics Statement

The studies involving human participants were reviewed and approved by Hangzhou Cancer Hospital. Written informed consent to participate in this study was provided by the participants’ legal guardian/next of kin. Written informed consent was obtained from the individual(s) for the publication of any potentially identifiable images or data included in this article.

## Author Contributions

Conceptualization: SZ. Project administration: YS. Writing – original draft: YS. Writing – review and editing: SZ. All authors contributed to the article and approved the submitted version

## Conflict of Interest

The authors declare that the research was conducted in the absence of any commercial or financial relationships that could be construed as a potential conflict of interest.
